# Genome Analysis of *Treponema pallidum* subsp. *pallidum* and subsp. *pertenue* Strains: Most of the Genetic Differences Are Localized in Six Regions

**DOI:** 10.1371/journal.pone.0015713

**Published:** 2010-12-29

**Authors:** Lenka Mikalová, Michal Strouhal, Darina Čejková, Marie Zobaníková, Petra Pospíšilová, Steven J. Norris, Erica Sodergren, George M. Weinstock, David Šmajs

**Affiliations:** 1 Department of Biology, Faculty of Medicine, Masaryk University, Brno, Czech Republic; 2 Department of Pathology and Laboratory Medicine, University of Texas-Houston Medical School, Houston, Texas, United States of America; 3 Department of Genetics, School of Medicine, The Genome Center, Washington University, St. Louis, Missouri, United States of America; Institut de Pharmacologie et de Biologie Structurale, France

## Abstract

The genomes of eight treponemes including *T. p. pallidum* strains (Nichols, SS14, DAL-1 and Mexico A), *T. p. pertenue* strains (Samoa D, CDC-2 and Gauthier), and the Fribourg-Blanc isolate, were amplified in 133 overlapping amplicons, and the restriction patterns of these fragments were compared. The approximate sizes of the genomes investigated based on this whole genome fingerprinting (WGF) analysis ranged from 1139.3–1140.4 kb, with the estimated genome sequence identity of 99.57–99.98% in the homologous genome regions. Restriction target site analysis, detecting the presence of 1773 individual restriction sites found in the reference Nichols genome, revealed a high genome structure similarity of all strains. The unclassified simian Fribourg-Blanc isolate was more closely related to *T. p. pertenue* than to *T. p. pallidum* strains. Most of the genetic differences between *T. p. pallidum* and *T. p. pertenue* strains were accumulated in six genomic regions. These genome differences likely contribute to the observed differences in pathogenicity between *T. p. pallidum* and *T. p. pertenue* strains. These regions of sequence divergence could be used for the molecular detection and discrimination of syphilis and yaws strains.

## Introduction

 Strains of *Treponema pallidum* subspecies *pallidum* (*T. p. pallidum*) are the causative agents of syphilis, whereas strains of *Treponema pallidum* subsp. *pertenue* (*T. p. pertenue*) cause yaws. These subspecies differ in their invasiveness and pathogenicity to humans. Although yaws, like syphilis, is a multi-stage disease, yaws is primarily restricted to skin and bone manifestations. Syphilis is a sexually transmitted disease affecting people worldwide, whereas yaws is transmitted by a direct skin contact predominantly in developing countries with a warm, humid climate. Moreover, unlike yaws strains, syphilitic treponemes can cross the placental barrier and infect the foetus. All of these differentiating characteristics reflect underlying differences in the genomic sequences of *T. p. pallidum* and *T. p. pertenue.*

In 1962, Fribourg-Blanc et al. [1] isolated a morphologically similar treponemal strain from a baboon (*Papio cynocephalus*) living in the West African Republic of Guinea. In this study, about 65% of the 111 Guinea baboons tested were seropositive for a treponemal infection [1]; however, no similar treponeme-reacting antibodies were found in more than 1300 sera taken from baboons from Kenya and Cambodia [2]. None of the seropositive baboons showed signs of infection clinically, but extracted bacteria were able to cause experimental hamster infection indicating that they were pathogenic [3]. Experimental inoculation of humans with the Fribourg-Blanc strain indicate that it is infectious to humans [4]–[6]. Other groups have also reported the occurrence of yaws in gorillas and chimpanzees [7].

The *T. p. pallidum*, *T. p. pertenue* and Fribourg-Blanc treponemes cannot be distinguished by morphology, protein content or physiology [3], [8]. Moreover, there is serological cross-reactivity between *T. p. pallidum* and *T. p. pertenue*, and the immune response in experimentally infected animals to the Fribourg-Blanc treponeme is indistinguishable from that to *T. p. pallidum.* These observations have been cited as evidence that syphilis and endemic treponematoses are caused by the same pathogen, and that the distinctive clinical manifestations of the diseases is a result of geographical, climate, host and other differences not related to genetic differences between these treponemes [9]–[12]. However, the simultaneous existence of syphilis and yaws, respectively, in neighboring urban and rural areas of equatorial Africa argues against this hypothesis [13].

There is an increasing amount of data showing that such genetic differences between *T. p. pallidum* and *T. p. pertenue* exist [14]. A genetic difference between *T. p. pallidum* Nichols and *T. p. pertenue* CDC 2575 was found in the gene TP1038 (*tpF1*) [15], and additional differences were identified in the 16S rRNA gene [16]. Sequence changes identified in the 5′- and 3′-flanking regions of TP0171 (*tpp*15) [17] differentiate *T. p. pallidum* from other tested treponemes, including *T. p. pertenue*, *T. p. endemicum* and the Fribourg-Blanc simian isolate. As shown by Centurion-Lara et al. [18], the *tprI* and *tprC* loci together with variable 5′- flanking regions of TP0171 (*tpp15*) can differentiate between *T. p. pallidum*, *T. p. pertenue*, *T. p. endemicum* and the unclassified simian isolate. The phylogenetic analysis of 6 *tpr* genes performed by Gray et al. [19] identified high levels of genetic variation between human treponemal subspecies when compared to observed variability within individual subspecies, supporting genetic separation of 3 treponemal subspecies into distinct entities.

A recent article by Harper et al. [20] mapped more than a dozen loci in the genomes of *T. p. pallidum, T. p. pertenue,* the Fribourg-Blanc isolate and other treponemal strains. They identified several nucleotide positions where all *T. p. pallidum* and *T. p. pertenue* strains investigated differed in a particular genome position. Moreover, the Fribourg-Blanc strain shared similar nucleotide changes as *T. p. pertenue*, indicating its close relationship to *T. p. pertenue* strains. However, a few nucleotide changes discriminated the Fribourg-Blanc strain from *T. p. pertenue*.

To further delineate the relationships between treponemal pathogens, we performed a systematic, whole genome comparison of four *T. p. pallidum* strains, three strains *T. p. pertenue*, and the Fribourg-Blanc simian isolate using whole genome fingerprints (WGF) and sequencing of divergent chromosomal regions.

## Results

### Whole genome fingerprinting (WGF) of *T. p. pallidum* strains (Nichols, SS14, DAL-1 and Mexico A), *T. p. pertenue* strains (Samoa D, CDC-2 and Gauthier) and the Fribourg-Blanc isolate

Four *T. p. pallidum* genomes, three genomes of *T. p. pertenue*, and the Fribourg-Blanc genome were amplified in 133 overlapping TPI amplicons, and the restriction patterns of these fragments were compared. The estimated genome sizes and differences in the restriction target sites (RTS) as well as the year and place of strain isolation are shown in Table 1. The previously published RTS data for the rabbit pathogen, *Treponema paraluiscuniculi* strain Cuniculi A [21] were also included in Table 1. In the Nichols genome, 223 *Bam*HI, 157 *Eco*RI, and 259 *Hin*dIII restriction sites were found. In all other genomes, the numbers of detected RTS were similar with only small differences, except of *T. paraluiscuniculi* genome, where the total number of different RTS was 190.

**Table 1:** Genome size and differences in restriction target sites (RTS) of *T. p. pallidum*, *T. p. pertenue, T. paraluiscuniculi* and Fribourg-Blanc strains.

Strain	Place and year of isolation	Reference	The source of the material	Estimated genome size (kb)	Number of missing RTS	Number of additional RTS	Total number of different RTS	Estimated genome sequence identity with Nichols (%)
Nichols	Washington, DC; 1912	[54]	Steven J. Norris, UT, Houston, TX, USA	1138.0 AE000520 1139.6^c^	-a	1^b^	1	100
DAL-1	Dallas; 1991	[41]	David L. Cox, CDC, Atlanta, GA, USA	1139.9	1	1	2	99.98
SS14	Atlanta; 1977	[55]	Steven J. Norris, UT, Houston, TX, USA	1139.5	3	5	8	99.92
Mexico A	Mexico; 1953	[45]	David L. Cox, CDC, Atlanta, GA, USA	1140.0	3	4	7	99.93
Samoa D	Western Samoa; 1953	[45]	Steven J. Norris, UT, Houston, TX, USA	1139.3	15	23	38	99.64
CDC-2	Akorabo, Ghana; 1980	[56]	David L. Cox, CDC, Atlanta, GA, USA	1139.7	17	22	39	99.63
Gauthier	Congo; 1960	[57]	Steven J. Norris, UT, Houston, TX, USA	1139.4	16	22	38	99.64
Fribourg-Blanc	Guinea; 1966	[3]	David L. Cox, CDC, Atlanta, GA, USA	1140.4	20	26	46	99.57
Cuniculi A	?	?	Steven J. Norris, UT, Houston, TX, USA	1133.4	96	94	190	98.21

a Altogether, 1773 RTS were tested in the Nichols genome.

b The additional *Acc*I RTS present in the Nichols genome resulted from the added *tprK*-like insertion in the intergenic region between TP0126–TP0127.

c The genome size was calculated from the published sequence [25] with addition of 7 repetitive sequence (60 bp) in genes TP0433–TP0434 and addition of the *tprK*-like insertion present in a part of the Nichols population [22].

**Figure 1:**
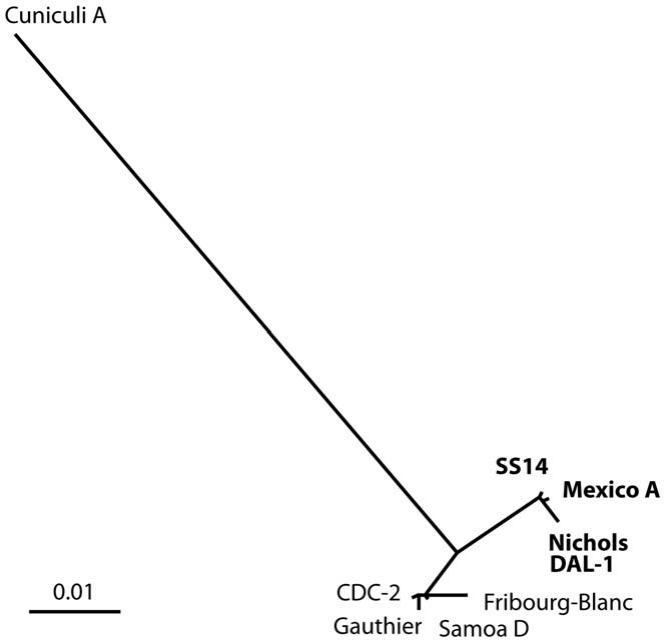
An unrooted tree showing the phypogenetic relationship of investigated genomes. An unrooted tree (Tree View) constructed from the binary RTS data illustrating the relatedness of individual genomes. In addition, we incorporated also RTS data for *T. paraluiscuniculi* strain Cuniculi A that were taken from the previously published work of Strouhal et al. [21]. Bar scale represents 0.01 restriction target site substitutions per tested RTS. *T. p. pallidum* strains causing syphilis are shown in bold.

The smallest genome was that of *T. paraluiscuniculi* strain Cuniculi A (1133.4 kb). The size of all other investigated genomes was very similar and fell into a range of 1139.3–1140.4 kb, representing a maximal genome size difference of 0.07%. The numbers of missing/additional restriction target sites were used as binary data for construction of unrooted tree illustrating the relatedness of individual genomes (Figure 1). An unrooted tree is presented; however, *T. paraluiscuniculi* represented a clear outlier.

The remaining genomes clustered into a *T. p. pallidum* cluster and a *T. p. pertenue* cluster that also contained the Fribourg-Blanc isolate (Figure 1). The *T. p. pallidum* cluster contained two pairs of related genomes including Nichols with DAL-1, and SS14 with Mexico A, respectively. Restriction target site analysis detected 1,773 individual RTS in the Nichols genome representing 10,636 bp. With the assumption that most differences in RTS were caused by single nucleotide changes, the estimated sequence similarity was calculated for all genomes (Table 1). The estimated genome sequence identity ranged between 99.57 and 99.98%; the similarity within *T. p. pallidum* strains when compared to similarity between *T. p. pallidum* and *T. p. pertenue* strains was substantially higher.

**Figure 2:**
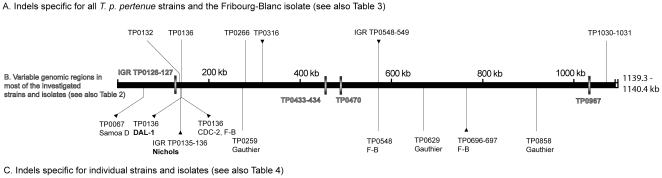
A schematic representation of genome changes found in *T. p. pallidum*, *T. p. pertenue* strains and Fribourg-Blanc isolate. **A** A schematic representation of indels found in all *T. p. pertenue* strains and the Fribourg-Blanc isolate but not found in any of the investigated *T. p. pallidum* strains (see also Table 3). Please note that TP0132 gene was not annotated in *pertenue* and Fribourg-Blanc strains. **B** Identified variable genomic regions in most of the investigated strains and isolates (see also Table 2). For more detailed structure of TP0126–TP0127 region see Figure 3, for details on TP0433–TP0434 locus, see [26]. **C** Indels specific for individual strains and isolates (see also Table 4). *T. p. pallidum* strains causing syphilis are shown in bold. Deletions are shown as vertical lines, insertions as lines with black triangles.

The WGF approach identified 15 genomic regions (TPI5B, TPI12A, TPI12B, TPI13, TPI21A, TPI21C, TPI25B-A, TPI32B, TPI34aa, TPI42A, TPI49, TPI55, TPI65B, TPI71A-C and TPI77) with detectable indels (Figure 2). Four regions showed variability in all investigated strains (in TPI intervals TPI12A, TPI32B, TPI34aa, and TPI71A-C). Six regions (in TPI intervals TPI12B, TPI13, TPI21C, TPI25B-A, TPI42A, and TPI77) showed changes in all *T. p. pertenue* and Fribourg-Blanc strains but none in *T. p. pallidum* genomes, whereas eight regions (in TPI intervals TPI5B, TPI13, TPI21A, TPI42A, TPI49, TPI55, and TPI65B) displayed changes in individual strains only. In the latter group, three such regions were found in the *T. p. pertenue* Gauthier strain (in TPI21A, TPI49, and TPI65B), one in Samoa D strain (in TPI5B), two in the Fribourg-Blanc isolate (in TPI42A, and TPI55), one in *T. p. pallidum* DAL-1 genome (in TPI13), and one in the Nichols genome (in TPI13).

### Variable genome regions present in all investigated strains

**Figure 3:**
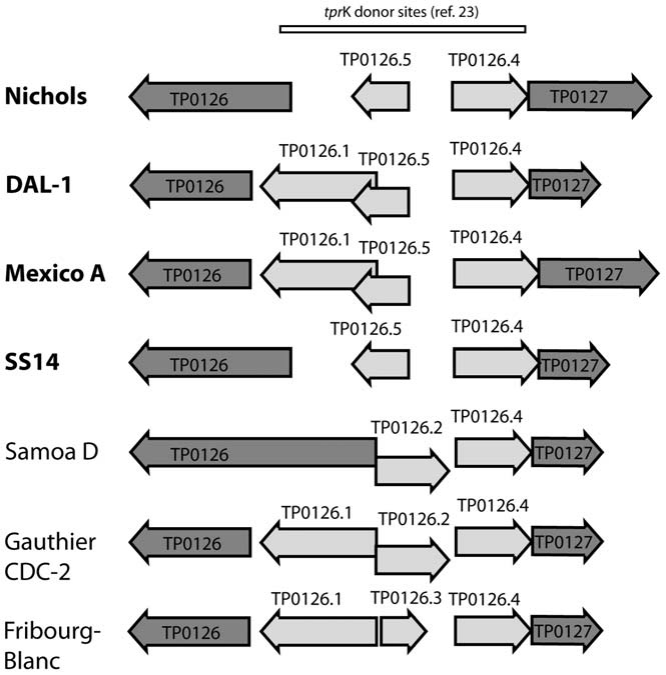
A schematic representation of the chromosomal region between TP0126 and TP0127. The newly annotated genes and the previously described gene conversion donor sites for the *tprK* variable (V) sequences [23] in the intergenic region between genes TP0126 and TP0127 are shown for each strain tested. *T. p. pallidum* strains causing syphilis are shown in bold.

The four genome regions showing variability found in most of the investigated strains are listed in Table 2 and depicted in Figure 2. In the intergenic region between genes TP0126 and TP0127, there was an insertion of *tprK*-like sequence of 1204 bp found in a subpopulation within the Nichols strain [22]. A similar insertion was found also in the DAL-1 genome. In the SS14 and Mexico A genomes, the *tprK*-like sequence was slightly longer (1255 bp). *T. p. pertenue* (Samoa D, CDC-2 and Gauthier) and the Fribourg-Blanc isolate showed a similar insertion of a *tprK*-like sequence of 1269 bp. The Nichols genome was the only one showing variability of this region on a strain population level. The occurrence of this *tprK*-like sequence in all isolates examined indicates that it was present in the common ancestor of these treponemal strains. The inserted *tprK*-like sequence is located in the 3′ flanking region of *tprD* that serve as a donor site for variable regions (V regions) of *tprK* gene [23]. Twenty of these donor sites (DS27–DS47, [23]) were localized in the inserted *tprK*-like sequence (between genes TP0126–TP0127) in all *pallidum* strains with the exception of DS31, which was altered in SS14 and Mexico A strains. In all investigated *pertenue* strains and in the Fribourg-Blanc isolate, only minor changes were found in the predicted donor sites including 1 nt change in DS38, DS40 and DS41, respectively. In addition, 6 nt changes were identified in DS39. Gene conversion-like mechanism between these donor sequences and the V regions of *tprK* gene was proposed [23]. In all investigated strains, gene prediction algorithms identified between TP0126-TP0127 loci 2 or 3 new genes encoding hypothetical proteins (Figure 3).

**Table 2:** Genome regions showing variability in most of investigated strains of *T. p. pallidum* (Nichols, SS14, DAL-1 and Mexico A), *T. p. pertenue* strains (Samoa D, CDC-2 and Gauthier), and in the Fribourg-Blanc isolate.

TPI interval/affected IGR or gene(s)/(coordinates following the Nichols genome [25])	Strain(s)	Detected indel		Total no. of repetitions	Putative gene function or sequence similarity	Characterization of hypothetical protein/predicted cellular localization^a^	GenBank accession no.
TPI12A IGR TP0126–TP0127 (148526–148527)	Nichols^b^, DAL-1,	insertion (1204 bp)			*tprK*-like sequence in *tprD* 3′ flanking region		HM585242, HM585259 Nichols HM585255 DAL-1
	SS14, Mexico A	insertion (1255 bp)					HM585243 SS14 HM585256, HM585257 Mexico A
	Samoa D, Gauthier, CDC-2, Fribourg-Blanc	insertion (1269 bp)					HM151364 Samoa D HM585245 Gauthier HM585244 CDC-2 HM585258 Fribourg-Blanc
TPI32B TP0433–TP0434 (461079–461499)	Nichols	insertion/deletion of repetitive sequences (60 bp per repetition)	insertion of 7 repetitions	14^c^	fusion of TP0433 and TP0434 to *arp* gene		-
	DAL-1		insertion of 7 repetitions	14			HM585240 DAL-1
	Mexico A		insertion of 9 repetitions	16			HM585249 Mexico A
	SS14		insertion of 7 repetitions	14			-
	Samoa D		insertion of 5 repetitions	12			HM585237 Samoa D
	Gauthier		insertion of 3 repetitions	10			HM585239 Gauthier
	CDC-2		deletion of 3 repetitions	4			HM585238 CDC-2
	Fribourg-BlancΔ		insertion of 8 repetition	15			-
TPI34aa TP0470 (497265–497688)	Nichols	insertion/deletion of repetitive sequences (24 bp per repetition)	-	17^d^	gene encoding conserved hypothetical protein	signal sequence, bacterial inner membrane	-
	DAL-1Δ		insertion of 10 repetitions	27			-
	Mexico AΔ		insertion of 9 repetitions	26			-
	SS14		deletion of 7 repetitions	10			-
	Samoa D		deletion of 5 repetitions	12			HM585241 Samoa D
	GauthierΔ		insertion of 8 repetitions	25			-
	CDC-2Δ		insertion of 20 repetitions	37			-
	Fribourg-BlancΔ		insertion of 5 repetitions	22			-
TPI71A-C TP0967 (1050281–1050282)	Mexico A, SS14	insertion (9 bp)			gene encoding hypothetical protein	bacterial cytoplasm	HM151373 Mexico A
	Samoa D	deletion (6 bp)					HM151370 Samoa D
	Gauthier, CDC-2, Fribourg-Blanc	insertion (12 bp)					HM151371 Gauthier HM151372 CDC-2 HM585251 Fribourg-Blanc

a The following algorithms were used for identification of sequence motifs and for prediction of cellular organization: SignalP, LipoP, CDD, Pfam, PSORT, and InterProScan.

b In the Nichols genome, insertion of 1204 bp exists only in its subpopulation [22].

c In the published Nichols genome sequence [25], only 7 tandem repetitions have been described in this region probably as a result of incorrect automated computer assembly. The correct number of repetitions in the Nichols strain is 14.

d In this region, the *T. p. pallidum* strains contain additional incomplete repetition (16 bp in length), *T. p. pertenue* strains have the same incomplete repetition of 18 bp length.

Δ The number of repetitions was estimated from PCR products visualized on agarose gels.

In the *arp* gene [24], variable number of repetitive sequences (60 bp in length) among investigated strains was found. *Arp* gene sequence corresponded to the TP0433 and TP0434 gene loci in the published genome sequence [25], and included most of the length of TP0433 and TP0434 genes. As shown by resequencing, the TP0433 and TP0434 genes are fused. The whole genome annotation of this chromosomal locus [25] as two genes resulted from sequencing error present in the published sequence [25]. In addition, 14 repetitions instead of 7 published in Fraser et al. [25] were found in the Nichols genome. The same number of repetitions was found in the SS14 and DAL-1 genome, whereas 16 repetitions were found in the Mexico A genome. The Fribourg-Blanc isolate showed 15 repetitions, a number similar to *T. p. pallidum* strains. In contrast, *T. p. pertenue* strains showed lower numbers of repetitive sequences ranging from 4 to 12. Moreover, the *arp* repeat region was not only variable in size, but also in the sequence of individual repeat units. In *T. p. pallidum* strains, 4 types (type I, II, III, II/III) of 60 bp-individual repeat sequences were found whereas in the *T. p. pertenue* strains, only 1 type of the repeat motif was identified [26]. In the Fribourg-Blanc isolate, sequentially identical repeat motifs were found [26], although the number of repeat units was similar to *T. pallidum* strains.

In the TPI34aa region, a variable number of repetitive sequences was found in gene TP0470 [21], [27]. The number of repetitions (24 bp in length) ranged between 10 and 37 (Table 2). In strains with 22 or more repetitions, the sequencing reads were not able to cover the entire region and ended inside one of the identical repetitive sequences. Because reads from both directions overlapped in repeats, the sequence of repeats was known. However, the exact number of repeats could not be deduced from the antiparallel sequence reads. Therefore, the number of repetitive motifs was estimated from the PCR products visualized on agarose gels. The hypothetical protein TP0470 with repetitions was predicted to be an inner membrane protein (Table 2).

In the region TPI71A-C (in hypothetical protein gene TP0967), Mexico A and SS14 strains showed an insertion of 9 bp, whereas Gauthier, CDC-2 and Fribourg-Blanc strains contained an identical 12 bp insertion. In contrast, a 6 bp deletion in this region was detected in the Samoa D genome. The sequence of TP0967 gene in the DAL-1 strain was identical to that of the Nichols sequence.

### Genome changes specific for *T. p. pertenue* strains and the simian Fribourg-Blanc isolate

We found six regions showing differences between all investigated *T. p. pallidum* and all tested *T. p. pertenue* strains (Figure 2). In all cases, the Fribourg-Blanc isolate showed changes similar to *T. p. pertenue* strains. These six regions contained 4 deletions and 2 insertions (Table 3). In the hypothetical protein gene TP0132, a deletion of 38 bp was observed in *T. p. pertenue* strains; in the Fribourg-Blanc isolate, sequence differences were dispersed over a broader region, reducing its length to 172 bp relative to the 210 bp region found in *T. p. pallidum* strains. The TP0132 gene was not annotated in the *T. p. pertenue* genomes due to these relative deletions, which result in frameshifts and thus shorter predicted protein products.

**Table 3 pone-0015713-t003:** Genome regions showing differences specific for *T. p. pertenue* strains (Samoa D, CDC-2 and Gauthier) and the simian Fribourg-Blanc isolate.

TPI interval/affected IGR or gene(s)/(coordinates following the Nichols genome [25])	Strain(s)	Detected indel	Putative gene function or sequence similarity	Characterization of hypothetical protein/predicted cellular localization^a^	GenBank accession no.
TPI12B TP0132 (152942–153151)	Samoa D, CDC-2, Gauthier, Fribourg-Blanc	several dispersed deletions (38 bp), 172 nt in this region remained	gene completely deleted		HM151364 Samoa D HM585245 Gauthier HM585244 CDC-2 HM585258 Fribourg-Blanc
TPI13 TP0136 (157368–157430)	Samoa D, Gauthier, CDC-2, Fribourg-Blanc	deletion (63 bp)	gene coding for fibronectin binding protein [28]		HM151364 Samoa D HM585245 Gauthier
(157457–157458)	CDC-2, Fribourg-Blanc	insertion (33 bp)			HM585244 CDC-2 HM585258 Fribourg-Blanc
TPI21C TP0266 (278334–278366)	Samoa D, CDC-2, Gauthier, Fribourg-Blanc	deletion (33 bp), substitution of 1 nt (278448) leading to cancellation of stop codon	partial deletion (11 aa) and elongation at C-terminus (5 aa) of gene coding for hypothetical protein	bacterial cytoplasm	HM165228 Samoa D HM165229 Gauthier HM165230 CDC-2 HM165231 Fribourg-Blanc
TPI25B-A TP0316 (331265–331266)	Samoa D, CDC-2, Gauthier, Fribourg-Blanc	insertion (635 bp) resulting in frameshift mutation	insertion of *tprI*-like sequence to *tprF* gene		HM585230 Samoa D HM585231 Gauthier HM585232 CDC-2 HM585233 Fribourg-Blanc
TPI42A IGR TP0548–TP0549 (593056–593057)	Samoa D, CDC-2, Gauthier, Fribourg-Blanc	insertion (52 bp)	prediction of a new hypothetical gene TP0548.1 (65 aa)		HM245777 Samoa D HM243496 Gauthier HM243495 CDC-2 HM585227 Fribourg-Blanc
TPI77 TP1030–TP1031 (1124020–1124396)	Samoa D, CDC-2, Gauthier, Fribourg-Blanc	deletion (377 bp) resulting in frameshift mutation	42 aa elongation of *tprL* at N-terminus		HM623430 Samoa D HM585235 Gauthier HM585236 CDC-2 HM585254 Fribourg-Blanc

a The following algorithms were used for identification of sequence motifs and for prediction of cellular organization: SignalP, LipoP, CDD, Pfam, PSORT, and InterProScan.

The gene coding for fibronectin binding outer membrane protein TP0136 [28] exhibited a 63 bp deletion in all non-*T. p. pallidum* strains. Gene TP0136 contains two nearly identical 96 nt-long repetitions [28] and the observed deletion was localized in the second one. In the genome of *T. p. pertenue* CDC-2 strain and of the Fribourg-Blanc isolate, an additional 33 bp insertion was found in this region. TP0136 protein was thus 10 amino acids shorter in the CDC-2 and in the Fribourg-Blanc strains and 21 amino acids shorter in the Samoa D and the Gauthier strains. However, the annotated lengths of the predicted proteins are 470 amino acids in Samoa D and Gauthier strains and 481 amino acids in CDC-2 and Fribourg-Blanc isolates because of the presence of additional sequence differences in the TP0136 gene.

All non-*T. p. pallidum* strains had a deletion of 33 bp in the TP0266 gene resulting in a 11 amino acid shorter hypothetical TP0266 protein and an insertion of 52 bp into the intergenic region between TP0548 and TP0549 genes. In the latter case, a new hypothetical gene TP0548.1 encoding a polypeptide with 65 amino acids in length was annotated.

An insertion of 635 bp in the *tprF* gene (TP0316) was found in all *T. p. pertenue* strains and also in the Fribourg-Blanc isolate. The insertion was sequentially similar to *tprI* and led to *tprF* elongation. A deletion of 377 bp in the region comprising genes TP1030 and TP1031 was found in the *T. p. pertenue* and Fribourg-Blanc strains and resulted in an elongation of the *tprL* (TP1031) gene.

### Genome regions with changes specific to individual strains

Eight strain-specific regions are listed in Table 4. Five out of the 8 strains investigated showed strain-specific genome differences (see also Figure 2). Three such regions were identified in the Gauthier strain comprising deletions in hypothetical genes of a variable length in the range between 9 bp and 302 bp. The Fribourg-Blanc isolate showed one 48 bp deletion in the hypothetical gene TP0548 and one insertion of repetitive sequence (430 bp in length) in the intergenic region (IGR) between TP0696 and TP0697 genes. A deletion of 303 bp in the hypothetical gene TP0067 was specific for the Samoa D genome. A specific 58 bp insertion was found in the DAL-1 strain in the gene coding for fibronectin binding outer membrane protein [28]. A deletion of 64 bp in the Nichols intergenic region between genes TP0135 and TP0136 was found in one Nichols subpopulation whereas the other one contained the longer version published previously [25]. Since all other investigated strains showed the shorter version in this region, the longer version published is specific for a Nichols subpopulation (see Table 4).

**Table 4 pone-0015713-t004:** Genome regions with changes specific to individual strains of *T. p. pallidum* (Nichols, SS14, DAL-1 and Mexico A), of *T. p. pertenue* strains (Samoa D, CDC-2 and Gauthier), and the Fribourg-Blanc isolate.

TPI interval/affected IGR or gene(s)/(coordinates according to the Nichols genome [25])	Strain(s)	Detected indel	Putative gene function or sequence similarity	Characterization of hypothetical protein/predicted cellular localization^a^	GenBank accession no.
TPI5B TP0067 (73405–73707)	Samoa D	deletion (303 bp)	gene coding for conserved hypothetical protein	TPR domain, bacterial cytoplasm	HM151365 Samoa D
TPI13 IGR TP0135–TP0136 (156488–156551)	Nichols^b^	insertion (64 bp)	-		-
TPI13 TP0136 (157949–158017)	DAL-1	insertion (58 bp) resulting in frameshift mutation, 67 nt in this region remained	gene coding for fibronectin binding protein [28], (452 aa)		HM585255 DAL-1
TPI21A TP0259 (270357–270365)	Gauthier	deletion (9 bp)	gene coding for hypothetical protein	LysM domain, bacterial inner membrane	HM151366 Gauthier
TPI42A TP0548 (591799–591846)	Fribourg-Blanc	deletion (48 bp)	gene coding for hypothetical protein		HM585227 Fribourg-Blanc
TPI49 TP0629 (686998–687299)	Gauthier	deletion (302 bp) resulting in frameshift mutation	gene coding for hypothetical protein (151 aa)	bacterial cytoplasm, signal sequence present in Nichols	HM151367 Gauthier
TPI55 IGR TP0696–TP0697 (764890–765321)	Fribourg-Blanc	insertion of repetitive sequence (430 bp)	**-**		HM151369 Fribourg-Blanc
TPI65B TP0858 (935500–935578)	Gauthier	continuous deletion (79 bp) resulting in frameshift mutation and small indels	gene coding for hypothetical protein (385 aa)	signal sequence, UPF0164 domain, bacterial inner membrane	HM151368 Gauthier

a The following algorithms were used for identification of sequence motifs and for prediction of cellular organization: SignalP, LipoP, CDD, Pfam, PSORT, and InterProScan.

b In the GenBank-deposited Nichols genome sequence, an insertion of 64 bp is included. All other investigated strains including subpopulation of the Nichols strain, have shorter version of this IGR.

### Changes identified by sequencing in the heterologous genome regions

TPI intervals showing length differences after restriction analysis were sequenced (corresponding accession numbers are shown in Table S2). Obtained sequences were compared to the corresponding ones in the reference Nichols genome. Altogether, 36 genes were sequenced in these heterologous regions in all 8 investigated genomes with the exception of 3 genes (TP0134, TP0315, TP0316) in the Mexico A strain and 3 additional genes (TP0433–434, TP1029) in the Fribourg-Blanc isolate. In 27 of these genes comprising TP0125, TP0128, TP0130, TP0133–134, TP0137–138, TP0265, TP0267–269, TP0430–432, TP0435–441, TP0549–553, TP1029, no major sequence changes (MSC) or frameshift mutations were found. Major sequence changes, defined as contiguous amino acid replacements comprising 10 and more residues or 15 and more dispersed amino acid replacements, and were observed in 15 genes (TP0126, TP0127, TP0129, TP0131, TP0132, TP0135, TP0136, TP0266, TP0315, TP0316, TP0433–TP0434, TP0548, TP1030 and TP1031) (see also Tables 2, 3 and 5). Three of these genes (TP0132, TP0135 and TP1030) were found to contain sequencing errors in the published Nichols genome [25]. In the TP0132, a false 1 nt deletion between coordinates 153123–153124 was found. Reannotation resulted in shortening of the hypothetical protein TP0132 from 69 to 64 amino acids. The reannotated TP0132 was similar to the corresponding genes in all other tested *T. p. pallidum* strains. However, in *T. p. pertenue* strains, gene TP0132 was not annotated (Table 3). Another false 1 nt deletion in the published Nichols genome was detected in the gene TP0135 between coordinates 155746–155747 leading to protein shortening (from 313 to 283 amino acids). Because of the sequencing protocol used, the published sequence of strain SS14 [27] contained the same sequence error in position 157003–157004. The corrected Nichols and SS14 sequences of the TP0135 gene showed only few nucleotide differences when compared to corresponding sequences in strains DAL-1, Mexico A, Samoa D, Gauthier, CDC-2 and Fribourg-Blanc. In the TP1030, two false 1 nt deletions were found between coordinates 1124003–1124004 and 1124188–1124189 in the Nichols genome, respectively. Moreover, in the published SS14 genome [27], a false 1 nt deletion between coordinates 1124003–1124004 of the Nichols genome (i.e. between SS14 coordinates 1125634–1125636) was found. Reannotation resulted in shortening of hypothetical protein TP1030 from 165 to 51 amino acids in both Nichols and SS14 genomes. The reannotated TP1030 gene was similar to the corresponding genes in the other *T. p. pallidum* strains. In *T. p. pertenue* strains, the 377 bp long deletion in this region resulted in deletion of the TP1030 gene (Table 3). Twelve remaining genes with MSC or frameshift comprising TP0126, TP0127, TP0129, TP0131, TP0266, TP0315, TP0316, TP0433–434, TP0548 and TP1031 are listed in Tables 2–5. Except for the *tprD* gene (TP0131), *fbp* gene (TP0136) [28], *tprF* (TP0316), *arp* gene (TP0433–TP0434) and *tprL* (TP1031), all 6 other genes (TP0126, TP0127, TP0129, TP0266, TP0315, and TP0548) coded for hypothetical proteins. At least four of them were predicted as inner or outer membrane proteins.

**Table 5:** Genome regions showing frameshifts and/or major sequence changes^a^ (MSC) of *T. p. pallidum* (SS14, DAL-1 and Mexico A), *T. p. pertenue* strains (Samoa D, CDC-2 and Gauthier), and the Fribourg-Blanc isolate when compared to the reference Nichols genome.

Gene	Strain	Detected frameshift or MSC (position according to the Nichols genome [25])	Protein change	Characterization of hypothetical protein/predicted cellular localization^b^	GenBank accession no.
TP0126^c^	DAL-1	1 nt deletion resulting in frameshift mutation (148340)	truncated hypothetical protein TP0126 (from 291 to 227 aa)	signal sequence present, bacterial inner membrane or periplasmic space (DAL-1, Mexico A, Gauthier, CDC-2, Fribourg-Blanc), bacterial cytoplasm (Nichols, Samoa D)	HM585255 DAL-1
	Mexico A				HM585256 Mexico A
	Gauthier				HM585245 Gauthier
	CDC-2				HM585244 CDC-2
	Fribourg-Blanc				HM585258 Fribourg-Blanc
TP0127 c	Mexico A	1 nt deletion resulting in frameshift mutation (148945)	truncated hypothetical protein TP0127 (from 229 aa to 222 aa)	DUF2715 domain, bacterial inner membrane (Mexico A)	HM585256 Mexico A
	DAL-1	2 nt deletion resulting in frameshift mutation (148944–148945)	truncated hypothetical protein TP0127 (from 229 aa to 126 aa)		HM585255 DAL-1
	SS14				-
	Samoa D				HM151364 Samoa D
	Gauthier				HM585245 Gauthier
	CDC-2				HM585244 CDC-2
	Fribourg-Blanc				HM585258 Fribourg-Blanc
TP0129	Samoa D	2 nt substitution (149875–149876)	premature stop codon resulting in 26-aa deletion at C-terminus of hypothetical protein TP0129 (from 158 to 132 aa)	bacterial cytoplasm	HM151364 Samoa D
	Gauthier				HM585245 Gauthier
	CDC-2				HM585244 CDC-2
	Fribourg-Blanc				HM585258 Fribourg-Blanc
TP0131	Mexico A	MSC and small indels (151122–152890)	truncated TprD (TP0131) protein (from 598 aa to 596 aa)		HM585256 Mexico A
	Samoa D				HM151364 Samoa D
	CDC-2				HM585244 CDC-2
	Fribourg-Blanc				HM585258 Fribourg-Blanc
TP0136	DAL-1	frameshift mutation (see Table 4) MSC and small indels (156887–158256)	truncated fibronectin binding protein TP0136 (from 495 aa to 452 aa)		HM585255 DAL-1
	SS14		(from 495 aa to 492 aa)		-
	Mexico A		(from 495 aa to 492 aa)		HM585257 Mexico A
	Samoa D		(from 495 aa to 470 aa)		HM151364 Samoa D
	Gauthier		(from 495 aa to 470 aa)		HM585245 Gauthier
	CDC-2		(from 495 aa to 481 aa)		HM585244 CDC-2
	Fribourg-Blanc		(from 495 aa to 481 aa)		HM585258 Fribourg-Blanc
TP0315	Samoa D	1 nt deletion resulting in frameshift mutation, MSC (330506)	elongation of conserved hypothetical protein TP0315 at C-terminus (from 215 aa to 270 aa)	DUF2715 domain, bacterial inner membrane (*T. p. pallidum* strains), bacterial outer membrane or periplasmic space (*T. p. pertenue* strains)	HM585230 Samoa D
	Gauthier				HM585231 Gauthier
	CDC-2				HM585232 CDC-2
	Fribourg-Blanc				HM585258 Fribourg-Blanc
TP0548	SS14	MSC and small indels (591822–592917)	elongation of treponemal conserved hypothetical protein TP0548 (from 434 aa to 438 aa)	bacterial inner membrane	-
	Mexico A		(from 434 aa to 438 aa)		HM585228 Mexico A
	Samoa D		shortening of treponemal conserved hypothetical protein TP0548 (from 434 aa to 432 aa)		HM245777 Samoa D
	Gauthier		(from 434 aa to 432 aa)		HM243496 Gauthier
	CDC-2		(from 434 aa to 432 aa)		HM243495 CDC-2
	Fribourg-Blanc		(from 434 aa to 418 aa)		HM585227 Fribourg-Blanc

a Major sequence changes were defined as continuous amino acid replacements comprising 10 or more residues or 15 and more dispersed amino acid replacements.

b The following algorithms were used for identification of sequence motifs and for prediction of cellular organization: SignalP, LipoP, CDD, Pfam, PSORT, and InterProScan.

c The reference Nichols strain was not resequenced in this region.

**Figure 4:**
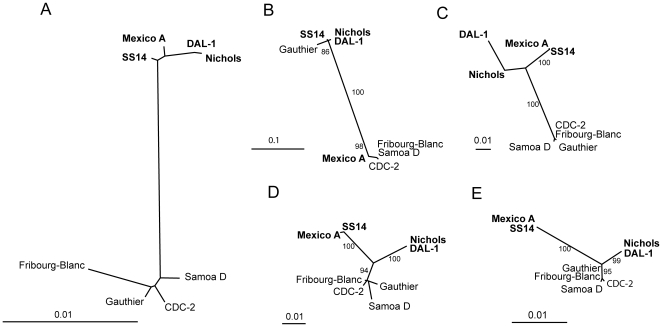
The unrooted trees constructed from sequences of genes showing major differences in strain clustering. **A** An unrooted tree constructed from the binary RTS data without Cuniculi A data. Bar scale represents 0.01 restriction target site substitutions per RTS. The unrooted trees constructed from sequences of 4 treponemal genes including TP0131, TP0136, TP0548, and TP1031 are shown in panel **B**, **C**, **D**, and **E**, respectively. Bar scale represents 0.01 nucleotide substitutions per site. Bootstrap values based on 1,000 replications are shown next to branches. *T. p. pallidum* strains causing syphilis are shown in bold.

To test whether the sequenced genes cluster the strains in a pattern similar to that obtained from the RTS analysis, trees showing phylogenetic relationships were constructed. Out of 36 sequenced genes, 8 genes showing more than 4 nucleotide replacements were selected and the corresponding sequences were used to construct unrooted trees. Four genes (TP0129, TP0132, TP0133, TP0266) showed a pattern very similar to the RTS tree (data not shown), whereas 4 genes (TP0131, TP0136, TP0548, and TP1031) differed from this tree (Figure 4). In the phylogenetic tree constructed from sequences of TP0131 (*tprD*), the *T. p. pertenue* strain Gauthier is closely related to *T. p. pallidum* strains whereas *T. p. pallidum* strain Mexico A is clustered with *T. p. pertenue* strains. In the other trees, constructed from sequences of TP0136, TP0548 and TP1031 genes, the sequence variability (shown as a length of tree branches) within *T. p. pallidum* strains is similar (or higher) than the sequence difference between *T. p. pallidum* and *T. p. pertenue* strains.

## Discussion

All investigated strains showed strikingly similar genome size varying only in 1.1 kb. The genome size showed no correlation with subspecies classification to *T. p. pallidum* and *T. p. pertenue*. Interestingly, the largest genome was found in the Fribourg-Blanc isolate, being 0.4 kb larger than the second largest genome of *T. p. pallidum* Mexico A. In contrast, the genome size of the *T. paraluiscuniculi* strain Cuniculi A (1133.4 kb, D. Šmajs, unpublished results) is considerably smaller, probably reflecting the different host specificities of *T. p. pallidum* and *T. paraluiscuniculi*. It is therefore clear that the genetic differences between *T. p. pallidum* and *T. p. pertenue* are in fact very subtle, although they are almost certainly responsible for the observed differences in pathogenesis in humans and experimentally infected animals. The differences in restriction sites, including additional and missing restriction target sites, clearly grouped *T. p. pallidum* strains into a separate cluster when compared to *T. p. pertenue* strains (Table 1, Figure 1). The *T. p. pertenue* group also contained the Fribourg-Blanc isolate, although it was more distantly separated from the *T. p. pertenue* strains. The close relationship between the Fribourg-Blanc treponemes and *T. p. pertenue* strains has been found also by Gray et al. [19], based on the phylogeny of *tprC* and *tprI* genes.

Analysis of restriction target sites provided another estimate of genome sequence similarity among the investigated strains to the Nichols genome. In this assessment, the assumption is made that mutation rates at the restriction sites occur at the same rate as the genomic DNA overall, and that there is no positive or negative selection with regard to restriction sites. Also, enzymes with a single sequence specificity were selected, so that a difference of a single base pair was sufficient to render a restriction site inactive. The analysis of 1,773 restriction sites in PCR amplicons in each of the 8 strains examined resulted in the representative sampling of 10,636 bp in each genome. The overall genome difference between *T. p. pallidum* and *T. p. pertenue* strains by this measure was only 0.36% to 0.37%, indicating an extreme sequence similarity between *T. p. pallidum* and *T. p. pertenue* despite the fact that strains belonging to both subspecies cause distinct and quite different human diseases. The close relatedness of Fribourg-Blanc isolate with *T. p. pertenue* strains suggests a possible transmission of a *T. p. pertenue* ancestor from African non-human primates to the human population (or vice versa). Interestingly, treponemes isolated from baboons appear to be well adapted to them and do not cause any clinical symptoms, but are pathogenic to hamsters [3]. The geographical regions with incidence of yaws overlap with the occurrence of simian treponemes [2]. In addition, this strain was able to cause human infection similar to yaws [4]–[6]. The syphilis and yaws-causing treponemes thus could originate from Africa as previously suggested by Livingstone [29].

Within the *T. p. pallidum* cluster, two subgroups of two strains each were observed, including Nichols and DAL-1 or SS14 and Mexico A strains, respectively. In 2006, Marra et al. [30] described the genetic diversity within *T. p. pallidum* strains in the intergenic region between TP0126 and TP0127 (see also Table 2). About 20% of clinical isolates in the USA did not contain a 51 bp insertion between these genes and were similar to the Seattle 81-4 strain, while 80% of them were similar to the SS14 strain [30]. This diversity within the *T. p. pallidum* cluster correlates with differences in other genomic regions (P. Pospíšilová, unpublished data), indicating that at least two genetically distinct groups of *T. p. pallidum* strains coexist in the human population.

The genomic regions showing variability in most of investigated strains (IGR TP0126-TP0127, *arp*, TP0470, TP0967) of *T. p. pallidum*, *T. p. pertenue* strains and the Fribourg-Blanc isolate were originally identified in the genome of *T. paraluiscuniculi* strain Cuniculi A [21]. This fact indicates that these regions might be variable in most other pathogenic treponemal strains and isolates. The *tprK*-like sequence inserted in the *tprD* 3′ flanking region was found in three versions of different lengths among the 8 investigated strains. Interestingly, in the Nichols genome, this insertion is present only in a smaller part of treponemal population [22], suggesting that this region could be deleted without markedly decreasing fitness. Indeed, it is possible that a deletion of this region occurred after passage to rabbits, which have been used for propagation of the Nichols strain for nearly a century. Two additional variable regions contain repetitive sequences with varying number of repetitions. In the *arp* gene [24], 14 repetitions (60 bp in length) were found in the Nichols genome. However, the published Nichols genome sequence [25], described only 7 tandem repetitions in this region probably as a result of incorrect automated computer assembly. Gain or loss of tandemly repeated sequences is likely to result from either slipped strand synthesis or recombination events and often has important biological functions, such as length variation in *Mycoplasma* coat lipoproteins [31]. In *Legionella pneumophila*, similar intragenic tandemly repeated sequences are often polymorphic and the number of repeats could reflect origin of the strains [32]. The observed difference in number of tandem repeats and the fact that the Arp protein is immunogenic [33] suggests a similar antigenic function. The *arp* repeat unit variability in *T. p. pallidum* strains is used as a part of molecular typing system differentiating at least 12 subtypes of *T. p. pallidum* [24]. Moreover, Liu et al. [33] and Harper et al. [26] classified *T. p. pallidum* Arp repeat motifs into 4 types (I, II, III, II/III) based on amino acid variations. Since *T. p. pertenue* strains contained identical repeat type (II) and differed from *T. p. pallidum* strains, variability in repeat types was correlated with a sexual transmission strategy [26].

Differences between *T. p. pallidum* and *T. p. pertenue* strains comprised several differences in *tpr* genes including the MSC and small indels in *tprD* in diverse strains, insertions in *tprF* and elongation of *tprL*. Centurion-Lara et al. [34] showed that several *tprD* alleles (*D, D2* and *D3*) exist among *T. p. pallidum* (*D, D2*) and *pertenue* (*D2, D3*) strains. The Mexico A strain contained the *D2* allele that is typical for *pertenue* strains with exception of the Gauthier strain (containing *D3* allele of *tprD*). These findings confirm the unusual clustering of the Mexico A and the Gauthier strains in the phylogenetic tree constructed from sequences of *tprD* locus (Figure 4B). Phylogenetic analyses performed by Gray et al. [19] revealed that *D2* allele at *tprD* is the ancestral allele and the other non-*D2* alleles are likely the result of 2 subsequent gene conversion events between the *tprD* and *tprC* loci, where the *tprC* serve as a donor. The observed difference in *tprD* (TP0131) cluster, when compared to phylogenetic tree obtained from RTS analysis, thus appears to result from gene conversion events [19]. On the other hand, increased variability within *T. p. pallidum* strains in TP0136, TP0548 and TP1031 (*tprL*), respectively, appears to result from positive selection of these loci in *pallidum* strains. The *tpr* genes are found in several strains of *Treponema pallidum* and also in *T. paraluiscuniculi*, and their paralogous proteins are sequentially related to the major outer sheath protein (Msp) of *Treponema denticola* [35]. The *tpr* genes are heterogeneous both within and between the *T. pallidum* subspecies and strains examined [34], [36], [37]. Although the precise role of *tpr* genes remains unknown, there is increasing evidence that the Tpr proteins are involved in pathogenesis and/or immune evasion. TprK protein induces a strong immune response [36], [38], [39], and the variable regions of TprK form targets for specific antibodies [40]. Since *tprF* is considerably shorter in *T. p. pallidum* strains and contains – when compared to *T. p. pertenue* strains and to Fribourg-Blanc isolate – a frameshift mutation, it is likely that *tprF* is either not functional or has an altered function in *T. p. pallidum* strains. Although *tprL* in either its shortened or elongated forms does not have a recognizable signal sequence in the corresponding protein sequence, these differences in amino acid sequence could result in altered cellular functions. In summary, at least three of the 12 *tpr* genes (*tprD*, *F*, and *L*) are different in sequence or longer in *T. p. pertenue* strains. We postulate that the longer versions represent ancient (original) *tpr* versions present in *T. p. pertenue* strains and in Fribourg-Blanc isolate.

The observed differences in the TP0136 gene coding for an antigenic fibronectin binding protein [28] could result in variations in the binding specificity and affinity of the *T. p. pertenue* versions of TP0136 lipoprotein that could possibly impact the pathogenic properties of *T. p. pertenue* strains. Interestingly, the fast-growing pathogenic strain DAL-1 [41] has a 58 bp insertion in this gene resulting in a frameshift and hence a major protein sequence change in this antigen. Three additional differences between *T. p. pertenue* and *T. p. pallidum* strains included changes in 2 hypothetical genes (TP0132, TP0266) and one in the IGR TP0548-TP0549. However, biological consequences of these differences remain unknown. Several hypothetical proteins encoded by the genes listed in Tables 2–5 are predicted inner or outer membrane proteins indicating their possible role in transport and antigenic properties of treponemes. Although no signal sequences were identified in the TprL and TP0548, these proteins were recently predicted as rare outer membrane proteins [42], favoring the latter function.

All the detected differences found in the tested genomes could be used for their molecular identification in clinical samples isolated from patients. This is an important application for differentiation of *pallidum* and *pertenue* strains, especially for African children [43] and pregnant women [44], where serological cross-reactivity of syphilis- and yaws-causing treponemes complicates the clinical diagnostics of both diseases. However, these differences need to be verified in a larger set of strains before they could be used as a reliable discriminative target between yaws- and syphilis-causing agents.

It has been proposed previously that syphilis, yaws, and the other endemic treponematoses are all caused by the same organism, and that climate or cultural differences result in differences in manifestations and transmission patterns. However, syphilis and yaws have distinctive lesions and patterns of pathogenesis, and typically do not provide full immunologic cross protection in experimental animal infections [14], [45]. The data presented in this study indicate that the genomes of *T. p. pallidum* and *T. p. pertenue* are very similar in both genome size and structure. Furthermore, the simian Fribourg-Blanc isolate is closely related to *T. p. pertenue* strains, suggesting that yaws may be a primate treponematosis rather than a distinctly human disease [3], [7]. Most of the genetic differences represent relatively simple genetic changes that occurred during the evolution of these genomes, and it is likely that these minor differences, representing less than 0.4% of the genome of these organisms, have given rise to the distinct infection patterns observed in syphilis and yaws. Unfortunately, because none of the *T. pallidum* subspecies nor *T. carateum* have been cultured continuously in vitro, directed mutagenesis of the divergent loci to determine their potential roles in pathogenesis is not currently possible. However, continued accumulation of genomic information, coupled with functional studies of the proteins encoded by these loci, may shed light on the important determinants involved in the microevolution of the pathogenic treponemes.

## Materials and Methods

### Isolation of *T. p. pallidum*, *T. p. pertenue* and Fribourg-Blanc chromosomal DNA

*T. p. pallidum* Nichols and SS14 as well as *T. p. pertenue* Samoa D and Gauthier chromosomal DNA was prepared as described by Fraser et al. [25] from treponemes obtained from experimentally infected rabbits. Treponemes were purified by Hypaque gradient centrifugation as described previously [25], [46]. DAL-1 and Mexico A (*T. p. pallidum* strains), CDC-2 (*T. p. pertenue* strain) and the unclassified Fribourg-Blanc simian isolate were obtained from David L. Cox (CDC, Atlanta, USA) as a rabbit testicular tissue containing treponemal cells (Table 1). To separate treponemes from the eukaryotic rabbit testicular cells, samples were briefly centrifuged at 100×g for 5 min to sediment these cells. The DNA of these samples was amplified from cells using QIAGEN Whole Genome Amplification REPLI-g Kit (Qiagen, Hilden, Germany) according to manufacturer's instructions.

### Whole genome fingerprinting

Whole genome fingerprinting was performed as described previously [21], [47]. The complete nucleotide sequence of the *T. p. pallidum* strain Nichols (GenBank accession no. AE000520, [25]) was divided into 133 overlapping DNA regions (*Treponema pallidum* intervals - TPI) ranging between 801 and 21000 bp. The median and average lengths of these TP intervals were 9110 bp and 9590 bp, respectively. Each TP interval was amplified using the GeneAmp® XL PCR Kit (Roche Molecular Systems, Branchburg, NJ, USA) according to manufacturer's recommended protocol with two reagent mixes. The Lower reagent mix contained 6.6 µL of water, 6.0 µL of 3.3× XL Buffer II, 4.0 µL of 10 mM dNTP Blend, 2.4 µL of 25 mM Mg(OAc)_2_ Solution, 0.5 µL of primer F (100 nmol/L) and 0.5 µL of primer R (100 nmol/L). The primers used are shown in Table S1 (Supplementary Material). The Upper reagent mix contained 19.0 µL of water, 9.0 µL of 3.3× XL Buffer II, 1.0 µL of r*Tth* DNA Polymerase XL and 1.0 µL of DNA template. The PCR reactions were performed on GeneAmp® PCR System 9700 (Applied Biosystems, Foster City, CA, USA) and started with initial denaturation step (94°C for 60 sec), continued by 16 cycles with temperature changing from 94°C (15 sec) to 65°C (10 min), followed by 12 cycles with temperatures 94°C for 15 sec and 67°C for 10 min with increment of 15 sec to each following cycle. The final PCR step comprised 72°C for 10 min. All PCR products were digested with an initial set of three restriction enzymes, *Bam*HI, *Eco*RI and *Hin*dIII or their combinations. In the Nichols reference genome (GenBank accession no. AE000520), 223 *Bam*HI, 157 *Eco*RI, and 259 *Hin*dIII restriction sites were present. Additional digestions were performed with *Acc*I (144 restriction target sites), *Ava*II (2 sites), *Bsm*AI (2 sites), *Cla*I (90 sites), *Dpn*I (5 sites), *Eco*RV (180 sites), *Hin*fI (9 sites), *Kpn*I (101 sites), *Mlu*I (200 sites), *Mse*I (17 sites), *Nco*I (57 sites), *Nde*I (4 sites), *Nhe*I (15 sites), *Nsi*I (22 sites), *Rsr*II (5 sites), *Sac*I (23 sites), *Sap*I (39 sites), *Spe*I (6 sites), *Sph*I (64 sites), *Xba*I (38 sites), and *Xho*I (111 sites) to reduce the distance between adjacent restriction sites to less than 4 kb. All enzymes were obtained from New England Biolabs (NEB, Frankfurt am Main, Germany). Two amplicons (TPI32B and TPI34aa) out of the total 133 amplified chromosomal regions contained repetitive sequences and were further analyzed by amplification of small subregions of each amplicon (481 and 771 bp, respectively) to accurately estimate the number of repetitions in amplicons. The repetitive sequences of TPI32B (between coordinates 461079–461499 in the Nichols genome) were amplified using 32BrepF1 (5′-CGTTTGGTTTCCCCTTTGTC-3′) and 32BrepR1 (5′-GTGGGATGGCTGCTTCGTATG-3′) primers. The TPI34aa subregion containing repetitions (Nichols coordinates 497265 - 497688) was amplified with TPI34F4 (5′-GTCTTGTGCACATTATTCAAG-3′) and TPI34R5 (5′-CTTCGTGCAACATCGCTACG-3′) primers.

### DNA sequencing

XL PCR products showing length differences when amplified from different treponemal strains were used for DNA sequencing using the *Taq* DyeDeoxy Terminator Cycle Sequencing Kit (Applied Biosystems, Foster City, CA, USA). Oligonucleotide sequencing primers from a previous study [21] were supplemented with additional primers designed using the Primer3 software [48]. The LASERGENE program package (DNASTAR, Madison, WI, USA) was used to assembe the consensus sequences.

### Construction of phylogenetic trees

The software PAUP* 4b10 [49] and its graphical interface PaupUp 1.0.3.1. Beta [50] were used for construction of phylogenetic trees using both binary restriction target site (RTS) data and nucleotide sequences of sequenced regions. A distance analysis was applied for binary data (number of additional/missing RTS), and the corresponding tree was built using the neighbor joining algorithm. DNA sequences used for tree constructions were aligned using on-line available ClustalX software (http://www.clustal.org/) [51] and the Modeltest 3.7 [52] was used to identify the best model of nucleotide substitutions. Phylogenetic trees were constructed by maximum parsimony method from aligned sequences or by using a maximum likelihood method from the best model identified by Modeltest 3.7. The TreeView 1.6.6 software [53] was used for graphical presentations of corresponding trees.

### Nucleotide sequence accession numbers

The nucleotide sequences reported in this study were deposited in the GenBank under the accession numbers HM151364–HM151373, HM165228–HM165232, HM245777, HM243495–HM243496, HM585227–HM585259, HM623430.

## Supporting Information

Table S1 **The list of primers used for whole genome fingerprinting (WGF) of treponemal genomes.**
(XLS)

Table S2 **The list of sequenced treponemal TPI regions and the corresponding GenBank accession numbers.**
(XLS)

## References

[1] Fribourg-Blanc A, Niel G, Mollaret HH (1963). Note of Some Imunological Aspects of the African Cynocephalus. 1. Antigenic Relationship of Its Gamma Globulin with Human Gamma Globulin. 2. Guinean Endemic Focus of Treponematosis.. Bull Soc Pathol Exot Filiales.

[2] Fribourg-Blanc A, Mollaret HH, Niel G (1966). Confirmation serologique et microscopique de la treponemose du cynocephale de Guinee.. Bull Soc Pathol Exot Filiales.

[3] Fribourg-Blanc A, Mollaret HH (1969). Natural treponematotis of the African primate.. Primates Med.

[4] Medina R (1963). WHO Technical Report WHO/VDT/RES 63.64..

[5] Smith JL (1971). Neuro-ophthalmological study of late yaws. I. An introduction to yaws.. Brit J Vener Dis.

[6] Smith JL, David NJ, Indgin S, Israel CW, Levine BM (1971). Neuro-ophthalmological study of late yaws and pinta. II. The Caracas project.. Br J Vener Dis.

[7] Levréro F, Gattis S, Gautier-Hion A, Ménard N (2007). Yaws disease in a wild gorilla population and its impact on the reproductive status of males.. Am J Phys Anthropol.

[8] Norris SJ, Cox DL, Weinstock GM (2001). Biology of *Treponema pallidum*: correlation of functional activities with genome sequence data.. J Mol Microbiol Biotechnol.

[9] Hollander DH (1981). Treponematosis from pinta to veneral syphilis revisited: hypothesis from temperature determination of disease patterns.. Sex Trans Dis.

[10] Hollander DH, Turner TB (1954). The role of temperature in experimental treponemal infections.. Am J Syph.

[11] Hudson EH (1965). Treponematosis in perspective.. Bull World Health Org.

[12] Baker BJ, Armelagos GJ (1988). The origin and antiquity of syphilis.. Curr Anthropol.

[13] Hackett C (1963). On the origin of the human treponematoses.. Bull WHO.

[14] Antal GM, Lukehart SA, Meheus AZ (2002). The endemic treponematoses.. Microbes Infect.

[15] Noordhoek GT, Wieles B, van der Sluis JJ, van Embden JD (1990). Polymerase chain reaction and synthetic DNA probes: a means of distinguishing the causative agents of syphilis and yaws?. Infect Immun.

[16] Walker EM, Howell JK, You Y, Hoffmaster AR, Heath JD (1995). Physical map of the genome of *Treponema pallidum* subsp. *pallidum* (Nichols).. J Bacteriol.

[17] Centurion-Lara A, Castro C, Castillo R, Shaffer JM, Van Voorhis WC (1998). The flanking region sequences of the 15-kDa lipoprotein gene differentiate pathogenic treponemes.. J Infect Dis.

[18] Centurion-Lara A, Molini BJ, Godornes C, Sun E, Hevner K (2006). Molecular differentiation of *Treponema pallidum* subspecies.. J Clin Microbiol.

[19] Gray RR, Mulligan CJ, Molini BJ, Sun ES, Giacani L (2006). Molecular evolution of the tprC, D, J, K, G and J genes in the pathogenic genus *Treponema*.. Mol Biol Evol.

[20] Harper KN, Ocampo PS, Steiner BM, George RW, Silverman MS (2008). On the origin of the treponematoses: a phylogenetic approach.. PLoS Negl Trop Dis.

[21] Strouhal M, Šmajs D, Matějková P, Sodergren E, Amin AG (2007). Genome differences between *Treponema pallidum* subsp.. *pallidum* strain Nichols and *T. paraluiscuniculi* strain Cuniculi A. Infect Immun.

[22] Šmajs D, McKevitt M, Wang L, Howell JK, Norris SJ (2002). BAC library of *T. pallidum* DNA in *E. coli*.. Genome Res.

[23] Centurion-Lara A, LaFond RE, Hevner K, Godornes C, Molini BJ (2004). Gene conversion: a mechanism for generation of heterogeneity in the tprK gene of *Treponema pallidum* during infection.. Mol Microbiol.

[24] Pillay A, Liu H, Chen CY, Holloway B, Sturm AW (1998). Molecular subtyping of *Treponema pallidum* subspecies *pallidum*.. Sex Transm Dis.

[25] Fraser CM, Norris SJ, Weinstock GM, White O, Sutton GG (1998). Complete genome sequence of *Treponema pallidum*, the syphilis spirochete.. Science.

[26] Harper KN, Liu H, Ocampo PS, Steiner BM, Martin A (2008). The sequence of the acidic repeat protein (*arp*) gene differenciates veneral from nonveneral *Treponema pallidum* subspecies, and the gene has evolved under strong positive selection in the subspecies that causes syphilis.. FEMS Immunol Med Microbiol.

[27] Matějková P, Strouhal M, Šmajs D, Norris SJ, Palzkill T (2008). Complete genome sequence of *Treponema pallidum* ssp. *pallidum* strain SS14 determined with oligonucleotide arrays.. BMC Microbiol.

[28] Brinkman MB, McGill MA, Pettersson J, Rogers A, Matějková P (2008). A novel *Treponema pallidum* antigen, TP0136, is an outer membrane protein that binds human fibronectin.. Infect Immun.

[29] Livingstone FB (1991). On the origin of syphilis: an alternative hypothesis.. Curr Anthropol.

[30] Marra CM, Colina AP, Godornes C, Tantalo LC, Puray M (2006). Antibiotic selection may contribute to increases in macrolide-resistant *Treponema pallidum*.. J Infect Dis.

[31] Citti C, Kim MF, Wise KS (1997). Elongated versions of Vlp surface lipoproteins protect *Mycoplasma hyorhinis* escape variants from growth-inhibiting host antibodies.. Infect Immun.

[32] Coil DA, Vandersmissen L, Ginevra C, Jarraud S, Lammertyn E (2008). Intragenic tandem repeat variation between *Legionella pneumophila* strains.. BMC Microbiol.

[33] Liu H, Rodes B, George R, Steiner B (2007). Molecular characterization and analysis of a gene encoding the acidic repeat protein (Arp) of *Treponema pallidum*.. J Med Microbiol.

[34] Centurion-Lara A, Sun ES, Barrett L, Castro C, Lukehart SA (2000). Multiple alleles of *Treponema pallidum* repeat gene D in *Treponema pallidum* isolates.. J Bacteriol.

[35] Fenno JC, Muller KH, McBride BC (1996). Sequence analysis, expression, and binding activity of recombinant major outer sheath protein (Msp) of *Treponema denticola*.. J Bacteriol.

[36] Centurion-Lara A, Castro C, Barrett L, Cameron C, Mostowfi M (1999). *Treponema pallidum* major sheath protein homologue TprK is a target of opsonic antibody and the protective immune response.. J Exp Med.

[37] Centurion-Lara A, Godornes C, Castro C, Van Voorhis WC, Lukehart SA (2000). The *tpr*K gene is heterogeneous among *Treponema pallidum* strains and has multiple alleles.. Infect Immun.

[38] Morgan CA, Lukehart SA, Van Voorhis WC (2002). Immunization with the N-terminal portion of *Treponema pallidum* repeat protein K attenuates syphilitic lesion development in the rabbit model.. Infect Immun.

[39] Morgan CA, Molini BJ, Lukehart SA, Van Voorhis WC (2002). Segregation of B and T cell epitopes of *Treponema pallidum* repeat protein K to variable and conserved regions during experimental syphilis infection.. J Immunol.

[40] Morgan CA, Lukehart SA, Van Voorhis WC (2003). Protection against syphilis correlates with specificity of antibodies to the variable regions of *Treponema pallidum* repeat protein K.. Infect Immun.

[41] Wendel GD, Sanchez PJ, Peters MT, Harstad TW, Potter LL (1991). Identification of *Treponema pallidum* in amniotic fluid and fetal blood from pregnancies complicated by congenital syphilis.. Obstet Gynecol.

[42] Cox DL, Luthra A, Dunham-Ems S, Desrosiers DC, Salazar JC (2010). Surface immunolabeling and consensus computational framework to identify candidate rare outer membrane proteins of Treponema pallidum.. http://dx.doi.org/10.1128/IAI.00834-10.

[43] Julvez J, Michault A, Kerdelhue V (1998). Serologic studies of non-venereal treponematoses in infants in Niamey, Niger.. Med Trop (Mars).

[44] Wilson J, Mauger DG (1973). Syphilis in pregnancy supervening on yaws: case report.. N Z Med J.

[45] Turner TB, Hollander DH (1957). Biology of the treponematoses..

[46] Baseman JB, Nichols JC, Rumpp O, Hayes NS (1974). Purification of *Treponema pallidum* from infected rabbit tissue: resolution into two treponemal populations.. Infect Immun.

[47] Weinstock GM, Norris SJ, Sodergren E, Šmajs D, Brogden KA, Roth JA, Stanton TB, Bolin CA, Minion FC, Wannemuehler MJ (2000). Identification of virulence genes *in silico*: infectious disease genomics.. Virulence mechanisms of bacterial pathogens. 3rd ed.

[48] Rozen S, Skaletsky HJ, Krawetz S, Misener S (2000). Primer3 on the WWW for general users and for biologist programmers.. Bioinformatics Methods and Protocols: Methods in Molecular Biology.

[49] Wilgenbusch JC, Swofford D (2003). Inferring evolutionary trees with PAUP*.. Curr Protoc Bioinformatics Chapter 6: Unit 6.4.

[50] Calendini F, Martin JF (2005). PaupUp v1.0.3.1 A free graphical frontend for Paup* Dos software..

[51] Larkin MA, Blackshields G, Brown NP, Chenna R, McGettigan PA (2007). Clustal W and Clustal X version 2.0.. Bioinformatics.

[52] Posada D, Crandall KA (1998). MODELTEST: testing the model of DNA substitution.. Bioinformatics.

[53] Page RD (1996). TreeView: an application to display phylogenetic trees on personal computers.. Comput Appl Biosci.

[54] Nichols HJ, Hough WH (1913). Demonstration of *Spirochaeta pallida* in the cerebrospinal fluid.. JAMA-J Am Med Assoc.

[55] Stamm LV, Kerner TC, Bankaitis VA, Bassford PJ (1983). Identification and preliminary characterization of *Treponema pallidum* protein antigens expressed in *Escherichia coli*.. Infect Immun.

[56] Liska SL, Perine PL, Hunter EF, Crawford JA, Feelez JC (1982). Isolation and transportation of *Treponema pertenue* in golden hamsters.. Curr Microbiol.

[57] Gastinel P, Vaisman A, Hamelin A, Dunoyer F (1963). Study of a recently isolated strain of *Treponema pertenue*.. Ann Dermatol Syphiligr Paris.

